# A Critical Appraisal of Lipid Management in the Post-Statin Era: Comparison on Guidelines, Therapeutic Targets, and Screening in a Case-Based Framework of Lipid Management

**DOI:** 10.1016/j.jacadv.2025.101823

**Published:** 2025-06-25

**Authors:** Jared Spitz, Jaideep Patel, Anandita Agarwala, Garima Sharma, Anurag Mehta, Pradeep Natarajan, Khurram Nasir, Pamela Morris, Roger S. Blumenthal, Michael D. Shapiro

**Affiliations:** a Inova Schar Heart and Vascular, Inova Health System, Falls Church, Virginia, USA; b Ciccarone Center for the Prevention of Cardiovascular Disease, Johns Hopkins Hospital, Baltimore, Maryland, USA; c Center for Cardiovascular Disease Prevention, Baylor Scott and White Health Heart Hospital Baylor Plano, Plano, Texas, USA; d Pauley Heart Center, Virginia Commonwealth University, Richmond, Virginia, USA; e Center for Genomic Medicine and Cardiovascular Research Center, General Hospital, Boston, Massachusetts, USA; f Program in Medical and Population Genomics, Broad Institute of Harvard and MIT, Cambridge, Massachusetts, USA; g Department of Medicine, Harvard Medical School, Boston, Massachusetts, USA; h Division of Cardiovascular Prevention and Wellness, Houston Methodist DeBakey Heart and Vascular Center, Houston, Texas, USA; i Department of Cardiology, Medical University of South Carolina, Charleston, South Carolina, USA; j Division of Cardiology, Wake Forest Baptist Health, Winston-Salem, North Carolina, USA

**Keywords:** atherosclerotic cardiovascular disease, cholesterol, lipids, pharmacotherapy, prevention, statins, triglycerides

## Abstract

The role of low-density lipoprotein-cholesterol in the pathogenesis of atherosclerotic cardiovascular disease (ASCVD) is well established. Lipid management remains the cornerstone of addressing ASCVD. In addition to statin therapy, there is a large and growing number of nonstatin therapies available to manage elevated cholesterol levels. This expert panel seeks to review current international recommendations regarding lipid management. In addition, complex yet commonly encountered lipid-management cases are provided. Guidance on applying guideline-based recommendations as well as newer evidence to the evaluation and management of ASCVD-risk lipid management is then provided.

## Highlights

• Lipid management remains central to cardiovascular disease reduction. Many new lipid drugs have been developed.
• A review of contemporary lipid guidelines and suggestions to harmonize recommendations is needed.
• Complex case examples are useful for helping understand usage of guidelines and new evidence.

The role of low-density lipoprotein-cholesterol (LDL-C) in the pathogenesis of atherosclerotic cardiovascular disease (ASCVD) is well established.^1^^,^^2^ Lipid-lowering therapies (LLT) are a cornerstone in the management of ASCVD risk by causing significant reductions in LDL-C and adverse cardiovascular events.^3^^,^^4^ The growing arsenal of LLT, from statins to newer therapies like proprotein convertase subtilisin kexin type 9 (PCSK9) inhibitors and bempedoic acid, provides clinicians with a robust toolkit to tailor treatment based on patient-specific risk profiles.^5^^,^^6^ Statins remain the foundational therapy, backed by decades of evidence supporting their efficacy in both primary^4^^,^^7^ and secondary prevention of ASCVD.^5^^,^^8^ However, statin intolerance and the need for more aggressive LDL-C reduction in high-risk individuals have driven the development of nonstatin alternatives.^5^^,^^8^^,^^9^ These nonstatin therapies expand treatment options for those in need of greater LDL-C lowering or who cannot tolerate statins. In addition, they enhance LDL-C clearance and offer new opportunities for combination therapy, achieving greater reductions in LDL-C levels than monotherapy alone. As guidelines continue to evolve, integrating these therapies into clinical practice allows for more individualized approaches to lipid management to improve outcomes for patients at risk of ASCVD.

This review discusses current options for LLT and summarizes recent major guidelines for lipid management. Furthermore, it seeks to close the divide between evidence-based guidelines and the complex scenarios often encountered in everyday clinical practice, providing actionable guidance for evaluation and management of ASCVD risk. By interpreting current recommendations and emphasizing the role of newer therapies, this work aims to equip clinicians with the knowledge necessary to make personalized, informed treatment decisions to optimize patient outcomes (Central Illustration).

**Central Illustration:**
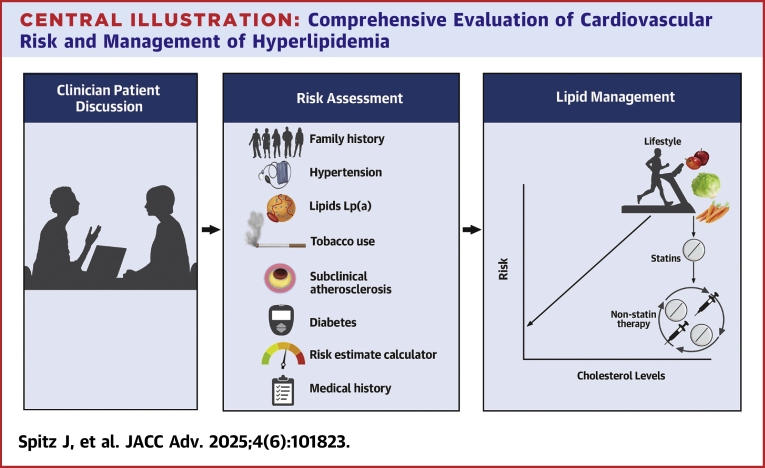
Comprehensive Evaluation of Cardiovascular Risk and Management of Hyperlipidemia The treatment of dyslipidemia starts with a clinician patient discussion to assess goals, potential, and options. The cornerstone of risk assessment is the use of clinically available risk-estimation calculators. Risk should further be personalized based on actors such as lipids, hypertension, diabetes, family history, and past medical history as well as imaging for subclinical atherosclerosis. Treatment begins with lifestyle modifications, and the degree of LDL-C lowering should be tailored to the risk of the patient with low LDL-C treatment thresholds for higher-risk patients. Statins remain the foundation of lipid-lowering therapy with additional medications added as needed based on achieved LDL-C levels, treatment goals, and adverse effects. Reassessment of therapy should be an ongoing process as should risk assessment and clinician patient discussions.

## Lipid-lowering therapies

A range of pharmacologic agents are available for lowering LDL-C levels, each playing a crucial role in reducing ASCVD risk.^5^^,^^10^ An overview of these medications is provided in Table 1^11^^,^^12^; treatments specifically for homozygous familial hypercholesterolemia (FH) and those undergoing clinical trials are not discussed.

**Table 1:** Lipid-Lowering Therapies^a^

Drug	Mechanism of Action	FDA Indications^b^	Common Usage/Notes	Lipid/Lipoprotein and Biomarker Changes	Reduction in Major Adverse Cardiovascular Events	Adverse Drug Effects
Statins	Inhibit HMG-CoA reductase	1. Primary prevention of ASCVD in those with increased cardiovascular risk 2. Established ASCVD 3. LDL-C reduction in primary hyperlipidemia 4. LDL-C reduction in heterozygous familial hypercholesterolemia 5. LDL-C reduction in homozygous familial hypercholesterolemia 6. Triglyceride reduction in hypertriglyceridemia and familial dysbetalipoproteinemia	1. First-line lipid-lowering therapy 2. Primary and secondary prevention indications 3. Cardiovascular outcome trials all on background statin therapy (except clear outcomes for bempedoic acid)	1. LDL-C reduction: ≥50% for high intensity; 30%-49% for moderate intensity 2. Modest triglyceride reduction 3. Modest HDL-C increase 4. Reduction in hs-CRP	22% reduction for every 38 mg/dL LDL-C reduction	1. Statin associated muscle symptoms 2. Elevated transaminases 3. Dysglycemia with risk of new-onset diabetes
Ezetimibe	Cholesterol absorption inhibitor	1. Adjunct therapy for primary hyperlipidemia (eg, HeFH) 2. Pediatric HeFH 3. Adult and pediatric HoFH 4. Homozygous sitosterolemia 5. In combination with fenofibrate to reduce LDL-C in mixed hyperlipidemia	1. Primarily used as adjunctive medication for additional LDL-C lowering when patient above treatment threshold	1. ∼15%-20% LDL-C reduction 2. Modest triglyceride reduction 3. Modest HDL-C increase	∼6% reduction	1. Diarrhea 2. Arthralgias
Bile acid sequestrants	Bind intestinal bile acids	1. Adjunct to diet for primary hyperlipidemia (including HeFH)	1. Rarely used in routine lipid management 2. Safe for lipid management during pregnancy	1. ∼15%-30% LDL-C reduction 2. Can cause marked hypertriglyceridemia 3. Can lower hemoglobin A1c	∼19% reduction	1. Gastrointestinal side effects 2. Contraindicated with hypertriglyceridemia
PCSK9 mAb (evolocumab and alirocumab)	Humanized monoclonal antibodies against PCSK9	Evolocumab: 1. Established ASCVD 2. Adjunct therapy for primary hyperlipidemia (eg, HeFH) 3. Pediatric HeFH 4. Adult and pediatric HoFH Alirocumab: 1. Established ASCVD 2. Adjunct therapy for primary hyperlipidemia (eg, HeFH)	1. Adjunct therapy in ASCVD 2. Adjunct therapy in HeFH 3. Adjunct therapy for additional LDL-C lowering when not at threshold per guidelines 4. Often used in statin intolerant individuals 5. Evolucumab and alirocumab often used interchangeably	1. ∼50%-60% reduction in LDL-C 2. Triglyceride reduction 3. ∼20%-30% reduction in Lp(a) 4. No hs-CRP reduction	∼15% reduction	1. Injection-site reaction 2. Nasopharyngitis 3. Influenza-like symptoms 4. Back pain (evolocumab)
Inclisiran	Small interfering RNA inhibitor of PCSK9	1. Established ASCVD 2. Adjunct therapy for primary hyperlipidemia (eg, HeFH) 3. High-risk primary prevention above LDL-C Threshold	1. Adjunct therapy in ASCVD 2. Adjunct therapy in HeFH 3. Adjunct therapy for additional LDL-C lowering when not at threshold per guidelines 4. Often used in statin intolerant individuals 5. Long-term cardiovascular outcome trials pending	1. ∼50% time averaged reduction in LDL-C 2. Triglyceride reduction 3. ∼20%-30% reduction in Lp(a) 4. No hs-CRP reduction	No cardiovascular outcome available (pending)	1. Injection-site reactions 2. Arthralgias
Bempedoic acid	ATP-citrate lyase inhibitor	1. Established ASCVD 2. Adjunct therapy for primary hyperlipidemia (eg, HeFH)	1. Often used in statin intolerant individuals although no FDA indication 2. Established ASCVD 3. Adjunct therapy in HeFH 4. Adjunct therapy	1. LDL-C reduction: ∼18% as monotherapy; ∼38% with fixed dose combination with ezetimibe; in combination with ezetimibe, ∼38% lowering 2. No reduction in Lp(a) 3. Reduction in hs-CRP	∼13% reduction	1. Hyperuricemia and gout 2. Tendon rupture: seen in those on high intensity statin, those with prior tendon injury, those taking corticosteroids and fluoroquinolone 3. Cholelithiasis
Icosapent ethyl	Not established^c^	1. Adjunct to maximally tolerated statin to reduce ASCVD risk in those with TG ≥150 mg/d L and established ASCVD or diabetes and additional cardiovascular risk factors 2. As an adjunct to diet in severe hypertriglyceridemia ≥500 mg/dL	1. Only prescription fish oil shown to reduce cardiovascular outcomes after optimizing LDL-C lowering 2. Unknown if TG lowering with this medication reduces pancreatitis	1. ∼15%-20% reduction in triglycerides 2. Dramatic reduction in hs-CRP 3. Minimal reduction in LDL-C, apolipoprotein B (event reduction greater than expected for LDL-C lowering)	∼25% reduction	1. Increased risk of atrial fibrillation or atrial flutter (3% vs 2% in placebo group)—most common in those with a history of atrial tachyarrhythmia 2. Increased risk of bleeding (12% vs 10% in placebo group; 3% serious bleeding)—greatest risk in those taking antiplatelet or anticoagulant therapy

ASCVD = atherosclerotic cardiovascular disease; FDA = Food and Drug Administration; HDL-C = high-density lipoprotein cholesterol; HeFH = heterozygous familial hypercholesterolemia; HMG-CoA = 3-hydroxy-3-methylglutaryl coenzyme A; HoFH = Homozygous familial hypercholesterolemia; hs-CRP = high-sensitivity C reactive protein; LDL-C = low-density lipoprotein cholesterol; TG = triglycerides.

a This table is limited to those medications in common usage for treatment of hyperlipidemia for primary and secondary prevention. Medications used exclusively for homozygous familial hypercholesterolemia (lomitapide, evinacumab) or that are still in clinical trial (eg, for Lp(a) elevation and severe hypertriglyceridemia) are not included.

b Different statins may have slightly varied FDA indications based on clinical trial data. However, this represents a composite of FDA indications for the generically available statins; specific FDA indications should be consulted as necessary.

c Believed to reduce hepatic triglyceride synthesis and packaging and/or increase triglyceride clearance from circulation.

The first point of emphasis is that statins remain the cornerstone of LLT. Statins inhibit HMG-CoA reductase, the enzyme that controls the rate-limiting step in cholesterol synthesis. A large body of evidence supports their use in both primary and secondary prevention of CVD.^4^^,^^5^ Despite their excellent safety profile, statin-associated muscle symptoms (SAMS) are a frequent cause of discontinuation in ∼7% to 10% of patients,^13^ leading to suboptimal LDL-C control and an increased risk of recurrent cardiovascular events.^14^

The second point to note is that in addition to statins, several nonstatin therapies have been developed and approved (Table 1). These agents offer additional options for patients requiring more substantial LDL-C reductions or those who are intolerant to statin therapy.^5^

The third key point is that combination therapy can provide additive effects. While doubling the dose of a statin results in diminishing returns (only an additional 6% reduction in LDL-C, such as if increasing atorvastatin from 40 mg-80 mg), combining a statin with nonstatin therapies can lead to an additional 20% to 50% reduction, depending on the agent used.^15^^,^^16^

## Brief review of cholesterol guidelines

The management of hypercholesterolemia has undergone significant evolution since the first major U.S. national cholesterol guidelines were introduced in 1988.^17^ This progress has been driven by the advent of statin therapy and numerous clinical trials demonstrating substantial reductions in cardiovascular events. A brief review of the most recent international guidelines is essential. These include the 2018 American Heart Association/American College of Cardiology (AHA/ACC) multisociety guidelines,^18^ with subsequent updates reflected in the 2021 and 2022 ACC Expert Consensus Decision Pathways (ECDPs) on hypertriglyceridemia^19^ and nonstatin therapies,^5^ respectively; the 2019 European Society of Cardiology (ESC) guidelines^20^; and the 2021 Canadian Cardiovascular Society (CCS) guidelines.^21^ Table 2^4^, ^5^, ^6^^,^^19^^,^^21^^,^^22^ provides an overview of the guidelines.

**Table 2:** Comparison of Recent International Cholesterol Statements

Recommendation	ACC/AHA^a^	ESC	CCS
Risk score used	Pooled Cohort Equation^b^	SCORE2/SCORE2-OP	FRS 10-y CHD risk
Risk stratification groups (and subdivisions)	1. Secondary prevention 1. Very high risk 2. High risk 3. LDL-C ≥190 mg/dL, not FH 4. Very high risk, LDL-C ≥190 mg/dL, and FH 2. Primary prevention, no DM: 1. Low risk (<5% 10-y risk) 2. Borderline risk (5%-<7.5% 10-y risk) 3. Intermediate risk (≥7.5%-<20% 10-y risk) 4. High risk (≥20% 10-y risk) 3. Primary prevention, DM: 1. Presence of diabetes risk enhancing factors 2. Absence of diabetes risk enhancing factors 4. Primary prevention, LDL-C ≥190 mg/dL	1. Very high risk 1. Clinical or imaging evidence of ASCVD 2. DM with target organ damage 3. Severe CKD (eGFR <30 mL/min/1.73 m^2^) 4. FH with ASCVD or other major risk factor 5. ≥10% 10-y risk 2. High-risk 1. TC >310 mg/dL, LDL-C >190 mg/dL, BP ≥180/110 mm Hg 2. FH without other risk factors 3. DM without target organ damage; ≥10 y of DM or other risk factor 4. Moderate CKD (eGFR 30-59 mL/min/1.73 m^2^) 3. Moderate risk 1. ≥1-<5% 10-y risk 2. T1DM <35 y old or T2DM <50 y old with DM <10 y and no other risk factors 4. Low risk (<1% 10-y risk)	1. Low risk (<10% 10-y risk) 2. Intermediate risk 1. 10%-19.9% 10-y risk 2. LDL-C ≥135 mg/dL or non-HDL-C ≥155 mg/dL or apolipoprotein B ≥105 mg/dL 3. Men ≥50 and women ≥60 y of age with additional risk factors 3. High risk (≥20% 10-y risk) 4. ASCVD
Definition of very high risk	Limited to secondary prevention: 1. History of multiple major ASCVD events OR 2. A major ASCVD event and multiple high-risk conditions Major ASCVD: • Recent ACS (within 12 mo) • History of MI (other than above ACS) • History of ischemic stroke • Symptomatic peripheral arterial disease High-risk conditions: • Age ≥65 y • DM • HTN • CKD • Heart failure • Current smoking • Heterozygous FH • History of CABG or PCI • Persistently elevated LDL-C ≥100 mg/dL despite maximal statin and ezetimibe	Not limited to secondary prevention: 1. Documented ASCVD, either clinical or unequivocal on imaging 2. Diabetes (with target organ damage OR 3 major risk factors OR type 1 DM >20 y) 3. CKD with eGFR <30 mL/min/1.73 m^2^ 4. A calculated score ≥10% for 10-y risk of fatal CVD 5. FH with ASCVD or another major risk factor	1. Recent ACS 2. Clinically evidence of ASCVD and a. DM or metabolic syndrome b. Polyvascular disease c. Symptomatic PAD d. Recurrent MI e. MI in previous 2 y f. Previous CABG g. LDL-C ≥100 mg/dL or heterozygous FH h. Lp(a) ≥60 mg/dL or 120 nmol/L High-risk conditions for primary prevention: • CKD • DM in patients >40 y or >30 y and with >15 y duration of diabetes or microvascular events • Abdominal aortic aneurysm >3.0 cm or AAA surgery
Risk-enhancing factors	1. Family history premature ASCVD 2. Persistent LDL-C >160 mg/dL 3. Elevated biomarkers: persistent TG ≥150 mg/dL, hs-CRP ≥2 mg/dL, Lp(a) >50 mg/dL, ApoB >130 mg/dL 4. ABI <0.9 5. Comorbidities: CKD not on dialysis, metabolic syndrome, inflammatory disease (eg, RA, psoriasis), HIV 6. Female-specific factors (preeclampsia, premature menopause) 7. South Asian ancestry 8. Diabetes-specific risk factors: long duration, albuminuria, eGFR <60 mL/min/1.73 m^2^, retinopathy, neuropathy, ABI <0.9	1. Family history premature ASCVD 2. Elevated biomarkers (ApoB, Lp(a), TG, CRP, albuminuria) with no cutoffs given 3. Carotid or femoral plaque on ultrasound: ABI <0.9 or >1.4 4. Comorbidities: chronic immune-mediated inflammatory disorders, HIV, major psychiatric disorders, atrial fibrillation, LVH, CKD, OSA, NAFLD 5. Psychosocial factors: social deprivation, inactivity, psychological stress	1. Family history of premature ASCVD 2. Abdominal obesity 3. Physical inactivity 4. Psychosocial factors 5. Excessive alcohol consumption 6. Coronary artery calcium score >0 Agatston units 7. Biomarkers: hs-CRP ≥2.0 mmol/L; Lp(a) ≥50 mg/dL (≥100 nmol/L) 8. Sex-specific conditions: Gestational hypertension, pre-eclampsia, eclampsia
Role of CAC	1. Risk modifier in select borderline and intermediate-risk patients when risk uncertain after a 10-y risk assessment 2. If CAC ≥1,000 AU, ≥50% LDL-C lowering and LDL-C <70 mg/dL; may consider high-intensity statin, ezetimibe, and PCSK9 inhibitor 3. If CAC ≥100 AU or ≥75th percentile, consider moderate- or high-intensity statin and LDL-C <70 mg/dL; ezetimibe can be considered 4. If CAC 1-99 AU and <75th percentile, consider moderate intensity statin and LDL-C <100 mg/dL; may increase to high-intensity statin 5. If CAC = 0 AU, can defer statin unless diabetes, LDL-C ≥190 mg/dL, smoker, family history premature CVD; repeat 3-5 y 6. If significant subclinical atherosclerosis previously documented or incidentally found, consider starting high-intensity statin and LDL-C <70 mg/dL	1. Risk modifier in low- or moderate-risk patients 2. If CAC>100 AU, reclassify as high risk and treat for LDL-C <70 mg/dL 3. No mention of downgrading risk based on CAC = 0 AU	1. Risk modifier in intermediate risk patients 2. Risk modifier in select low-risk patients: family history, FH, elevated Lp(a) 3. In intermediate-risk patients: • If CAC >100 AU, indication for intensive risk factor modification • If CAC >300 AU = very high risk category • No mention of downgrading risk based on CAC = 0 AU 4. In high-risk patients (FH, Lp(a), poorly controlled risk factors) • If CAC = 0 AU, “aggressive risk factor modification” • If CAC >0 AU, “strong rationale for adherence” to aggressive risk factor modification including lipid-lowering therapy or treatment intensification
Primary Prevention Treatment	1. No DM: a. Low risk 1. Lifestyle changes b. Borderline risk 1. Lifestyle 2. Consider moderate-intensity statin c. Intermediate risk 1. Moderate intensity statin for 30%-49% LDL-C lowering and LDL-C <100 mg/dL; consider increasing statin dose if >100 mg/dL d. High risk 1. High-intensity statin for ≥50% LDL-C lowering and LDL-C <70 mg/dL; consider adding ezetimibe if >70 mg/dL 2. DM: a. If <7.5% risk, no diabetes specific risk factors^c^, or CAC, moderate intensity statin for 30%-49% LDL-C reduction and LDL-C <100 mg/dL; increase statin dose if needed b. If ≥7.5%-19%, diabetes-specific risk factors, or CAC, high-intensity statin recommended c. If ≥20% risk, high-intensity statin for ≥50% LDL-C reduction and LDL-C <70 mg/dL; may consider addition of ezetimibe	1. Primary prevention very high-risk with FH a. High intensity statin for ≥50% LDL-C lowering and LDL-C <55 mg/dL. b. Ezetimibe followed by PCSK9 inhibitor recommended if LDL-C ≥55 mg/dL (class 1 for both drugs) 2. Primary prevention very high-risk without FH a. High intensity statin for ≥50% LDL-C lowering and LDL-C <55 mg/dL. b. Ezetimibe (class 1) followed by PCSK9 inhibitor (class 2b) recommended if LDL-C ≥55 mg/dL 3. Primary prevention high risk a. High intensity statin for ≥50% LDL-C lowering and LDL-C <70 mg/dL. b. Consider ezetimibe if LDL-C 70 mg/dL. No comment on additional therapy. 4. Primary prevention moderate risk a. High intensity statin for ≥50% LDL-C lowering and LDL-C <100 mg/dL b. Consider Ezetimibe if LDL-C >100 mg/dL. No comment on additional therapy. 5. Primary prevention low risk 1. High intensity statin for ≥50%	1. Low risk a. Lifestyle changes b. If LDL-C ≥5 mmol/L (∼190 mg/dL), use maximally tolerated statin; if LDL-C <50% reduced or ≥2.5 mmol/L (∼100 mg/dL), add ezetimibe or PCSK9 inhibitor 2. Intermediate risk a. If LDL-C ≥3.5 mmol/L (∼135 mg/dL), use maximally tolerated statin b. If LDL-C remains ≥2 mmol/L (∼77 mg/dL), consider ezetimibe 3. Diabetes or CKD a. Use maximally tolerated statin b. If LDL-C remains ≥2 mmol/L (∼77 mg/dL), consider ezetimibe
	3. LDL-C ≥190 mg/dL: a. High intensity statin for ≥50% LDL-C reduction and LDL-C <100 mg/dL b. Consider ezetimibe and/or PCSK9 mAb if LDL-C >100 mg/dL c. May consider bempedoic acid or inclisiran	a. LDL-C lowering and LDL-C <116 mg/dL. b. Consider ezetimibe if LDL-C >116 mg/dL. No comment on additional therapy.	
Secondary prevention treatment	1. ASCVD not at very high risk: a. High-intensity statin for ≥50% LDL-C lowering and LDL-C <70 mg/dL b. If LDL-C >70 mg/dL 1) Consider adding ezetimibe 2) Consider adding or replacing with PCSK9 mAb 3) May consider bempedoic acid or inclisiran 2. ASCVD at very high risk a. High-intensity statin for ≥50% LDL-C lowering and LDL-C <55 mg/dL b. If LDL-C >55 mg/dL 1) Consider adding ezetimibe and/or PCSK9 mAb 2) May consider bempedoic acid or inclisiran 3. ASCVD not at very high risk with LDL-C ≥190 mg/dL a. High-intensity statin for ≥50% LDL-C lowering and LDL-C <70 mg/dL b. If LDL-C >70 mg/dL 1) Consider adding ezetimibe 2) Consider adding or replacing with PCSK9 mAb c. May consider bempedoic acid or inclisiran	1. Secondary prevention at very high risk a. High intensity statin for ≥50% LDL-C lowering and LDL-C <55 mg/dL. b. Ezetimibe followed by PCSK9 inhibitor recommended if LDL-C >55 mg/dL (class I for both drugs) 2. ASCVD with recurrent event within 2 y a. High intensity statin for ≥50% LDL-C lowering and LDL-C <40 mg/dL b. Ezetimibe followed by PCSK9 inhibitor recommended if LDL-C >40 mg/dL	1. ASCVD a. Maximally tolerated statin b. If LDL-C 1.8- 2.2 mmol/L (66-85 mg/dL), consider ezetimibe ± PCSKi c. If LDL-C> 2.2 mmol/L (85 mg/dL), consider PCSK9 inhibitor ± ezetimibe
	4. ASCVD at very high risk with LDL-C≥ 190 mg/dL a. High-intensity statin for ≥50% LDL-C lowering and LDL-C< 55 mg/dL b. If LDL-C >55 mg/dL 1) Consider adding ezetimibe and/or PCSK9 mAb 2) May consider bempedoic acid or inclisiran		
Triglyceride management	1. Adults without DM or ASCVD and TG 150-499 mg/dL a. Low 10-y risk: diet b. Borderline-intermediate 10-y risk: initiate or intensify statin c. High 10-y risk: initiate or intensify to high-intensity statin 2. Adults with DM and no ASCVD and TG 150-499 mg/dL a. Maximize statin therapy b. If <50 y old or ≥50 with no risk enhancing factors, continue statin therapy c. If ≥50 y old with risk enhancing factors, consider icosapent ethyl 3. Adults with ASCVD and TG 150-499 mg/dL a. Maximize statin therapy b. If LDL-C ≥100 mg/dL, consider nonstatin therapy c. If LDL-C <70 mg/dL, consider icosapent ethyl d. If LDL-C 70-99 mg/dL, consider hybrid LDL-C and TG-lowering approach	In high-risk (or greater) patients, jf TG ≥1.5-5.6 mmol/L (∼135 mg/dL-499 mg/dL), consider icosapent ethyl 2 g BID in addition to statins	In secondary prevention, if TG ≥1.5-5.6 mmol/L (∼135 mg/dL-499 mg/dL), consider icosapent ethyl 2 g BID

ABI = Ankle-Brachial Index; ACC = American College of Cardiology; AHA = American Heart Association; ASCVD = atherosclerotic cardiovascular disease; AU = Agatston unit; CAC = coronary artery calcium; CKD = chronic kidney disease; DM = diabetes mellitus; eGFR = estimated glomerular filtration rate; FH = familial hypercholesterolemia; HIV = Human Immunodeficiency Virus; hs-CRP = high-sensitivity C reactive protein; LDL-C = low-density lipoprotein cholesterol; RA = rheumatoid arthritis; TG = triglycerides.

a Based on the 2022 ACC Expert Consensus Decision Pathway if updated from 2018 ACC/AHA guideline.

b PREVENT equation is not currently incorporated into guidelines or consensus statements.

c Duration (≥10 years for T2DM or ≥20 years for T1DM), albuminuria, CKD, retinopathy, neuropathy, ABI <0.9.

Two of the most significant differences across these guidelines are the approach to risk classification and treatment.^3^

The AHA/ACC and CCS guidelines make an initial distinction between primary and secondary prevention; the AHA/ACC guidelines also separately provide recommendations for those with diabetes and severe hypercholesterolemia (LDL-C ≥190 mg/dL).^18^^,^^21^ In contrast, the ESC guidelines delineate patients from those with established ASCVD to those with low-calculate 10-year risk; however the guideline does make note of primary and secondary prevention in discussions of treatment.^20^

In regard to treatment and nonstatin use, the ESC overall is more aggressive in LDL-C treatment thresholds and recommends LDL-C ≤55 mg/dL for all secondary and very-high-risk primary prevention patients.^20^ In contrast, the AHA/ACA guidelines reserve this for very-high-risk secondary prevention patients.^5^^,^^18^ Similarly, treating to LDL-C ≤70 mg/dL is recommended for a wide group of patients in ESC guidance.^20^

In terms of therapeutic options, the biggest divergence is on the use of PCSK9 inhibitors. These are recommended for secondary and select primary prevention patients in ESC guidelines^20^ although it is noteworthy that evidence of benefit in nonsecondary prevention and non-FH patients is lacking although clinical trials are ongoing.^23^ In contrast, use is limited in the ACC ECDP to secondary-prevention patients and those with likely FH.^5^

## Future outlook and key considerations

Recognizing the evolving landscape of lipid management, several key areas should be considered in future updates of the AHA/ACC guidelines.

### Quantitative risk assessment

One of the most significant issues in future risk assessment is the role of the newly developed PREVENT (AHA Predicting Risk of CVD Events) equations.^24^ Derived from a much larger data set than the Pooled Cohort Equations (PCE), PREVENT eliminates race and ethnicity as variables and incorporates additional inputs such as hemoglobin A1c, urine albumin, estimated glomerular filtration rate, and zip code as a surrogate for the Social Deprivation Index. Validation studies suggest that PREVENT offers a more accurate risk-prediction model than PCE. Furthermore, it expands the age range for risk prediction, beginning at 30 years old, and provides both 10- and 30-year estimates for CVD, ASCVD, and heart failure risk.^24^ However, comparative analyses show that PREVENT generally provides lower risk estimates than PCE, classifying a greater number of individuals as lower risk.^25^^,^^26^ This trend has substantial implications—modeling suggests that over 15 million individuals previously recommended for statin therapy may no longer meet traditional risk thresholds (10-year risk >7.5%), potentially leading to an additional 107,000 myocardial infarctions or ischemic strokes over a 10-year period.^25^^,^^26^ However, the critique of the PREVENT equations assumes that the current risk thresholds used with the PCE will remain unchanged. If future guidelines adjust these thresholds to account for the lower risk estimates generated by PREVENT, concerns regarding this reclassification may be mitigated. This remains an area for ongoing evaluation and underscores the need for careful consideration in future updates to clinical practice guidelines.

### Lipid testing

Beyond conventional recommendations for lipid testing, selective evaluation of additional lipoproteins should be considered. First, Lp(a) screening should be given strong consideration, considering a high prevalence of ∼20% in the general population. Lp(a) is a widely recognized independent risk factor for ASCVD and has been listed as a risk-enhancing factor in U.S.-based guidelines.^22^ However, many major societies recommend universal Lp(a) testing,^20^^,^^21^^,^^27^ and the time has come for ACC/AHA guidelines to follow suit. However, while trends are shifting toward universal screening, it is worth nothing that there are no specific Lp(a)-lowering treatments currently available. While there is guidance regarding contemporary treatment, specific outcome trials of Lp(a)-lowering drugs are ongoing.^27^ While it is noted as a risk-enhancing factor in AHA/ACC Cholesterol Guidelines,^18^ there is a need for clearer guidance on apolipoprotein B (ApoB) testing, given its critical role in the pathophysiology of ASCVD and as a risk factor, especially in patients with metabolic syndrome and high levels of triglycerides. European and Canadian guidelines already recommend ApoB measurement, and the National Lipid Association's recent guidance in this area is encouraging.^28^ As with Lp(a), it warrants mention that LLT outcome trials have not used ApoB as a target of treatment, and therefore, recommendations in this regard are based on secondary analyses.^28^

### Subclinical atherosclerosis evaluation

**Figure 1:**
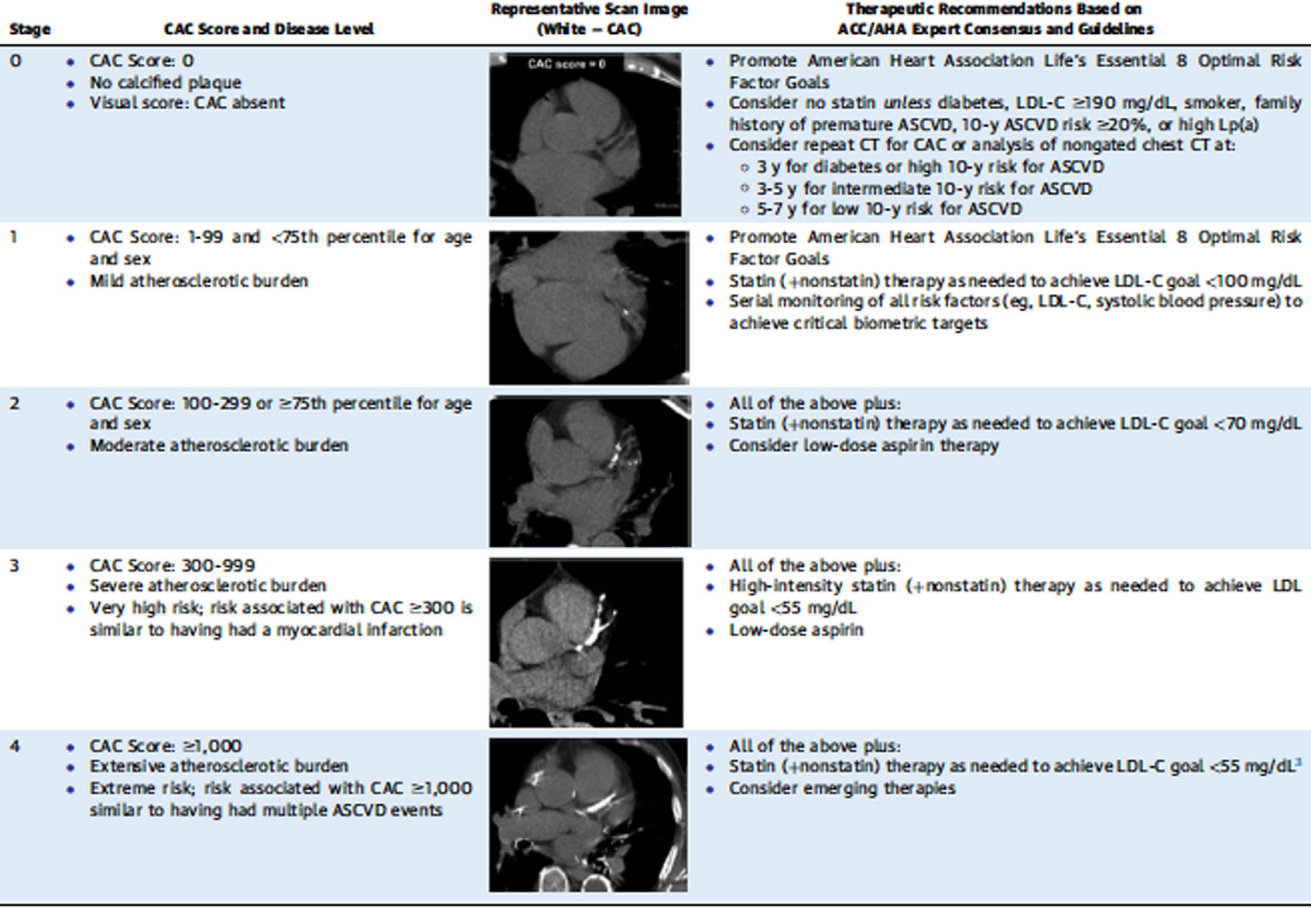
Proposed Coronary Artery Calcium Staging Guide to Therapy Used with permission of the publisher.^30^

Subclinical atherosclerosis assessment, particularly through coronary artery calcium (CAC) scoring, plays an increasingly important role in risk stratification for borderline and intermediate-risk patients.^5^^,^^18^ More recent recommendations outlined in the 2022 ACC ECDP provide specific treatment guidance based on the CAC score.^5^ However, a recent analysis of the multinational CONFIRM (Coronary CT Angiography Evaluation for Clinical Outcomes: An International Multicenter Registry) sought to evaluate at what CAC level individuals without a history of ASCVD should be treated as aggressively as those who survived an ASCVD event. This analysis suggests that a CAC score of 300 Agatston units (AU) portends a risk for individuals that is equivalent to that for secondary prevention patients and thus may warrant the same level of preventive pharmacotherapy.^29^ Furthering this work and building on the recommendations of the ECDP, one proposal from Maron et al^30^ suggests further subclassifying CAC scores between 100 to 299, 300 to 999, and ≥1,000 AU, rather than simply using a threshold of ≥100 (Figure 1). Treating high CAC scores more aggressively, akin to secondary prevention, is a very reasonable approach.

### Treatment recommendations

Future guidelines should harmonize ACC/AHA treatment approaches with those of contemporary international guidelines. For example, the ESC recommendation that secondary prevention patients should target an LDL-C <55 mg/dL is a more stringent goal than current ACC/AHA guidelines and should be considered.^20^ Of note, AHA/ACC guidelines and ACC ECDP rely mainly on randomized controlled trial evidence. The ESC guidelines emphasize randomized controlled trials but take into account the totality of evidence, including nonrandomized trials and epidemiologic studies. Therefore, the more aggressive recommendations are based on data beyond clinical outcome trials.^5^^,^^18^^,^^20^ In addition, patients with high plaque burden (CAC ≥300) should be treated to a similar LDL-C target.^29^^,^^30^ In alignment with ESC and CCS guidelines, PCSK9 inhibitors should be considered in treatment algorithms for high-risk primary prevention patients, not solely for secondary prevention or those with LDL-C ≥190 mg/dL.^20^^,^^21^^,^^31^

There is also merit in considering pharmacotherapy for low- and moderate-risk patients with persistently elevated LDL-C despite lifestyle modification. This recommendation is supported by data from the Cholesterol Lowering in Intermediate-Risk Persons without Cardiovascular Disease (HOPE-3) trial^32^ and recent imaging studies demonstrating early, yet reversible, plaque formation in patients with dyslipidemia. The forthcoming PRECAD (Prevent Coronary Artery Disease) trial will provide further insights into this issue.^33^

The 2021 ACC ECDP on the Management of ASCVD Risk Reduction in Patients With Persistent Hypertriglyceridemia should also remain central to future guideline updates,^19^ particularly in light of the REDUCE (Cardiovascular Risk Reduction with Icosapent Ethyl for Hypertriglyceridemia) trial results,^11^ which were published after the 2018 guidelines. This trial established the benefit of icosapent ethyl, a highly purified eicosapentaenoic acid ethyl ether derivative, in reducing cardiovascular risk in those with persistent hypertriglyceridemia. However, there has been controversy about the use of a mineral oil placebo in REDUCE-IT^34^^,^^35^ and conflicting data from the STRENGTH (Effect of High-Dose Omega-3 Fatty Acids vs Corn Oil on Major Adverse Cardiovascular Events in Patients at High Cardiovascular Risk) trial,^36^ which used corn oil and a different omega-3 fatty acid preparation. Despite this, the ACC ECDP, ESC, and CCS recommend icosapent ethyl for residual hypertriglyceridemia.^19^, ^20^, ^21^ In addition, the ACC/AHA Cholesterol Guidelines should offer specific recommendations for managing individuals with elevated Lp(a), in line with recent National Lipid Association guidance, which advocates initiating or intensifying therapy in those with LDL-C ≥70 mg/dL.^27^

### Special populations

Several specific populations require further attention in future guidelines:

• Younger adults: Although atherosclerosis is known to begin early in life,^37^^,^^38^ 10-year risk estimates tend to underrepresent the risk of younger adults due to age's significant impact on risk calculations.^39^ While PREVENT begins risk estimates at age 30,^24^ current guidelines provide little direction on managing dyslipidemia in younger adults.^18^^,^^38^^,^^40^ Future guidelines should incorporate 30-year risk estimates for this population and consider persistent LDL-C elevations (eg, LDL-C >130 mg/dL despite lifestyle changes) as a risk-enhancing factor.^32^^,^^41^ Imaging studies have shown that subclinical atherosclerosis can develop even at LDL-C levels below 130 mg/dL.^37^^,^^38^^,^^40^
• Women of reproductive age: This group warrants special consideration, particularly given the rising prevalence of cardiovascular risk factors in younger women, such as age of menarche, polycystic ovarian syndrome, and adverse pregnancy outcomes such as preeclampsia or gestational diabetes.^37^^,^^42^^,^^43^ Fewer than 10% of pregnant women currently maintain optimal cardiovascular health.^18^^,^^43^ While the 2018 guidelines mention increases in LDL-C and triglycerides during pregnancy, recommendations are largely limited to discontinuing statin therapy before pregnancy.^18^^,^^43^ Given the established relationship between adverse pregnancy outcomes and future ASCVD risk, more comprehensive guidance on lipid management in women of reproductive age is necessary.^18^^,^^43^^,^^44^ Recommendations should include prepregnancy dyslipidemia optimization, particularly in women with severe dyslipidemia such as FH or elevated Lp(a), and should incorporate recent National Lipid Association guidelines for managing lipid levels during and after pregnancy.^27^^,^^43^, ^44^, ^45^, ^46^
• South Asian populations: South Asian adults tend to have a higher risk of early and more aggressive ASCVD (nearly double compared to White populations), often at lower levels of traditional risk factors.^18^^,^^46^ While the 2018 AHA/ACC Cholesterol Guidelines include South Asian ancestry as a risk-enhancing factor,^18^^,^^46^ we believe further specificity is needed. South Asian adults are not a monolith with variation in risk based on country of origin. The prevalence of diabetes and hypertension is elevated and often underrecognized, and dyslipidemia patterns are that of lower LDL-C, higher triglycerides, and lower high-density lipoprotein cholesterol (HDL-C), emphasizing the importance of non-HDL-C and ApoB in risk assessment.^18^^,^^46^^,^^47^ There may also be a case for more aggressive LDL-C targets in high-risk South Asian patients,^46^^,^^47^ including consideration of early CAC screening for borderline- or intermediate-risk individuals, especially as available risk calculators typically underestimate ASCVD risk in this population.^7^^,^^46^^,^^47^
• Patients with HIV: The 2018 Cholesterol Guideline recognizes HIV as a risk-enhancing factor,^18^^,^^48^ but recent data from the Pitavastatin to Prevent Cardiovascular Disease in HIV Infection (REPRIEVE) trial show that low- to moderate-risk HIV-positive patients benefit from statin therapy.^7^^,^^12^^,^^18^^,^^48^ Future guidelines should include specific recommendations for primary prevention in HIV-infected individuals.

### Therapeutic inertia and combination therapy

Compelling evidence of therapeutic inertia in treating high-risk patients supports the consideration of initiating combination therapy (such as fixed-dose combination pills) for those presenting with an ASCVD event.^8^^,^^9^^,^^45^^,^^46^ Both European and US data consistently show that lipid-lowering intensity and achieved LDL-C fall short of guideline recommendations across high- and very-high-risk patients.^8^^,^^9^^,^^15^^,^^49^ Given the recognition that increasing the dose of statins yields diminishing returns on further LDL-C reduction,^43^ often at the cost of poor statin tolerance, there has been growing recognition of the role of combination LLT.^15^^,^^50^ Furthermore, as most nonstatin drug trials have had patients on background statin therapy, the majority of data already support combination therapy for achieving ever lowering treatment levels.^15^^,^^47^, ^48^, ^49^, ^50^ This also lends credence to the argument that the reduction in ASCVD events in lipid-lowering trials is driven by LDL-C lowering as opposed to properties unique to any given drug class, which is also supported by the Cholesterol Treatment Trialists.^4^ Indeed, the use of more moderate statin intensities in combination with nonstatin therapy (generally ezetimibe) has consistently shown good efficacy compared to high-intensity statin monotherapy.^15^^,^^51^

Cohort studies have shown that moderate-potency statins and ezetimibe can be superior in achieving recommended LDL-C levels and reducing cardiovascular events^17^^,^^52^^,^^53^; while it was an open-label, noninferiority trial, the long-term efficacy and safety of moderate-intensity statin with ezetimibe combination therapy vs high-intensity statin monotherapy in patients with ASCVD (RACING) trial demonstrated noninferiority of a moderate-intensity statin and ezetimibe in reducing a composite major adverse cardiovascular events endpoint.^50^^,^^54^ This has led to recommendations from one group of European providers to begin upfront combination therapy in those at high and very-high risk. The availability of fixed-dose combination medications in Europe will certainly facilitate this compared to the United States where our only clinically available fixed-dose combination pill is bempedoic acid and ezetimibe.

## Case-based framework

Recent guidelines and ECDPs provide the foundation for managing primary and secondary prevention in cardiovascular disease. However, clinical practice often presents challenging patient scenarios that do not fit neatly within current guideline frameworks. In the following sections, several cases are discussed to highlight how these guidelines can be applied to complex clinical situations.

## Primary prevention with CAC = 0 AU

A 57-year-old African American female with a history of hyperlipidemia, not currently on therapy, and with well-controlled hypertension on hydrochlorothiazide 25 mg daily presents for her annual primary care visit. Her fasting lipid panel reveals the following:

• LDL-C: 156 mg/dL
• HDL-C: 29 mg/dL
• Triglycerides: 170 mg/dL
• Total cholesterol: 219 mg/dL

Her blood pressure is 130/79 mm Hg, and her hemoglobin A1c is 4.6%. She is a never-smoker, and there is no family history of heart disease. Her primary care physician calculates her 10-year ASCVD risk using the PCE, resulting in a 10.6% estimated risk (PREVENT 10-year risk of CVD 7.1%, ASCVD 4.3%). The patient expresses hesitation about initiating statin therapy. To further refine her risk estimation, a CAC score is obtained, which returns a result of 0 AU.

### Discussion

Given this patient's intermediate 10-year ASCVD risk, as calculated by the PCE, and her CAC score of 0, the patient's risk can be further refined. Both the 2018 ACC/AHA cholesterol guidelines and the 2022 ACC ECDP provide specific guidance for cases where CAC scoring is used to refine risk stratification and guide therapy decisions, particularly when there is patient uncertainty about statin initiation.

For patients with a zero CAC score, considerable evidence suggests low short-term risk of cardiovascular events.^55^ Consensus recommendations support that in these cases, statin therapy may be safely deferred if there is absence of severe hyperlipidemia (LDL-C ≥190 mg/dL), diabetes, tobacco use, or family history of premature coronary artery disease. Recommendations support reassessing a CAC score at 3 to 5 years.^5^ This is similar to recommendations from the CCS guidelines although no specific guidance on treatment deferral for CAC of 0 is made.^21^ Notably, while the ESC guidelines note a low event rate with a CAC score of 0, no comment is made on the impact on treatment.^20^

In summary, for this patient, the CAC score of 0 shifts her risk profile into a low-risk category. Given the excellent short-term prognosis, statin therapy can be safely deferred with interval testing of her lipids and CAC score.

## Severe hypertriglyceridemia

A 44-year-old South Asian man with a history of prediabetes presents to establish care for persistent hypertriglyceridemia, which has been documented for the past 3 years. His most recent fasting triglyceride level is 506 mg/dL. Other relevant laboratory results include:

• LDL-C: 93 mg/dL
• HDL-C: 37 mg/dL
• Total cholesterol: 231 mg/dL
• Hemoglobin A1c: 6.3%

The patient reports a predominantly sedentary lifestyle and follows a traditional South Asian vegetarian diet rich in simple carbohydrates, such as white rice. He is a never-smoker and has no family history of heart disease. His body mass index (BMI) is 29.3 kg/m^2^, and his waist circumference is 43 inches. He has never experienced pancreatitis and is not currently taking any medications.

This patient presents with severe hypertriglyceridemia and prediabetes, 2 interrelated conditions that increase his risk of both cardiovascular disease and pancreatitis. The elevated triglycerides, coupled with his elevated fasting blood sugar and hemoglobin A1c, suggest a metabolic profile heavily influenced by his diet and lifestyle. Notably, secondary causes of hypertriglyceridemia include prediabetes, obesity (as defined by the World Health Organization's BMI cutoffs for South Asian populations),^56^ a carbohydrate-rich diet, and a sedentary lifestyle.^57^

### Discussion

For patients with hypertriglyceridemia in the primary prevention setting, lifestyle modifications represent the first-line intervention and are the cornerstone of management. Key recommendations include:

• Dietary changes: Reduce consumption of added sugars, limit total fat intake, and substitute simple carbohydrates with fiber-rich, complex carbohydrates. This approach addresses the patient's reliance on carbohydrate-rich foods such as white rice, which contributes to his hypertriglyceridemia.
• Physical activity: Encourage adherence to guideline-recommended physical activity, specifically aiming for at least 150 minutes per week of moderate-intensity aerobic exercise. Aerobic exercise has been shown to reduce triglycerides by about 11%, with resistance training contributing to insulin sensitization and being associated with a further 6% reduction in triglyceride levels. Weight loss should also be emphasized, given his elevated BMI and waist circumference, both of which are contributing factors to his metabolic risk.
• Alcohol abstinence: If applicable, abstaining from alcohol is important as it can significantly exacerbate hypertriglyceridemia, although this is not relevant to this patient since he is a never-smoker and presumably abstains from alcohol.^19^^,^^58^

Lifestyle interventions should be implemented over a period of 4 to 12 weeks, after which a repeat fasting triglyceride level should be measured to assess the response.^19^

**Figure 2:**
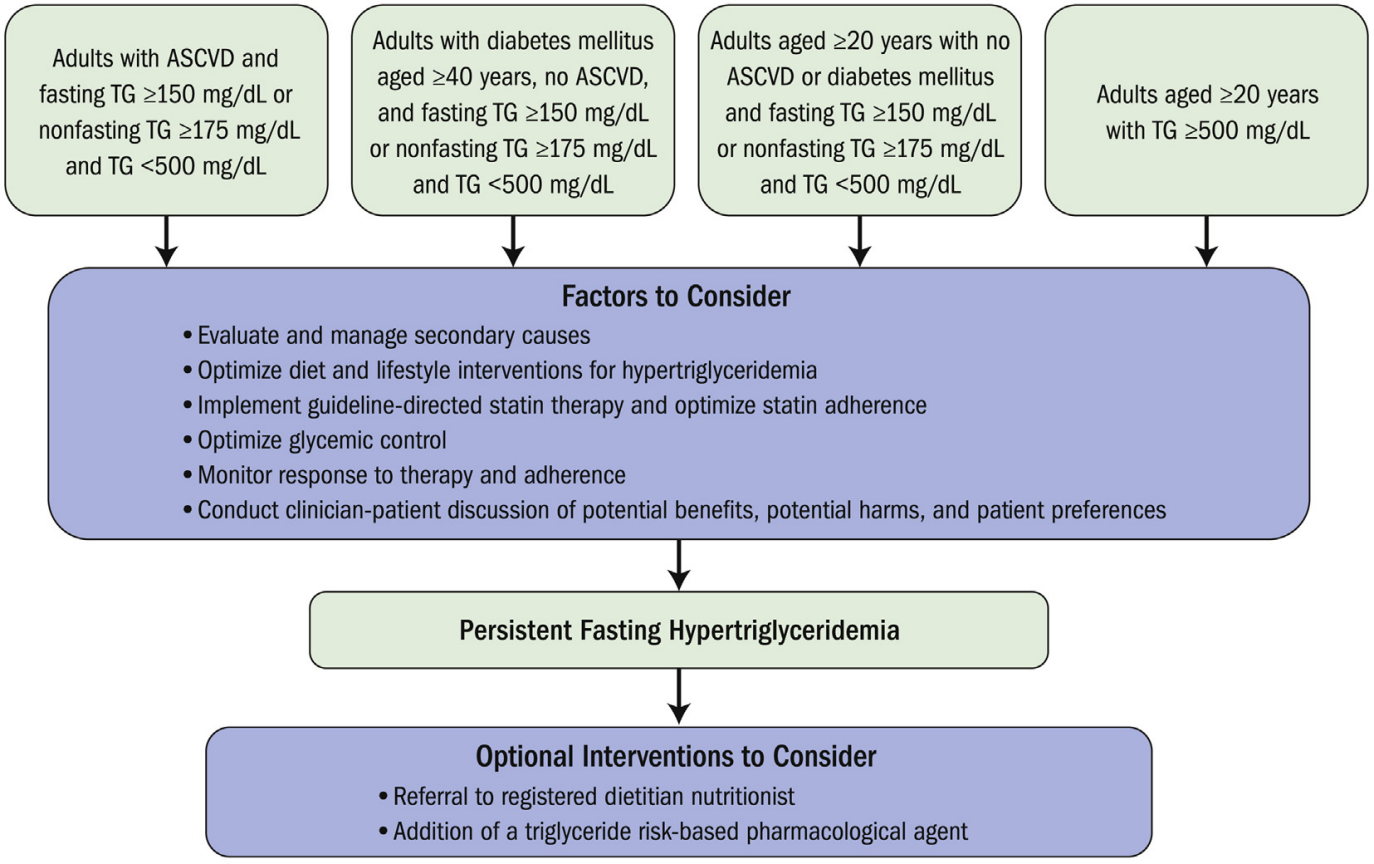
Overview of Triglyceride Management Adapted From the 2022 Expert Consensus Decision Pathway on the Management of ASCVD Risk Reduction in Patients With Persistent Hypertriglyceridemia Used with permission of the publisher.^19^ ASCVD = atherosclerotic cardiovascular disease; TG = triglycerides.

According to the 2021 ACC ECDP on the Management of ASCVD Risk Reduction in Patients With Persistent Hypertriglyceridemia, triglyceride levels above 500 mg/dL warrant targeted therapy to reduce the risk of pancreatitis, particularly if lifestyle modifications alone are insufficient. For this patient, pharmacologic options should be considered if triglycerides remain elevated after a period of lifestyle optimization^19^ (Figure 2).

• Very-low-saturated-fat diet: Lifestyle therapy is the cornerstone of triglyceride management as many cases of hypertriglyceridemia are secondary to suboptimal lifestyle and cardiometabolic disarray. In individuals with persistently elevated triglycerides >500 mg/dL despite optimization of lifestyle as mentioned earlier and glycemic control, further dietary restriction with a very-low-saturated-fat diet should also be considered, especially in patients without significant ASCVD or diabetes. This dietary approach can further support triglyceride lowering and reduce cardiovascular risk.^19^^,^^57^
• Fenofibrate or prescription omega-3 fatty acids: These agents can be considered to lower triglycerides and reduce the risk of pancreatitis in patients with persistently elevated levels (≥500 mg/dL) after lifestyle optimization and addressing secondary causes. Prescription-strength omega-3 fatty acids, such as icosapent ethyl or omega-3 acid ethyl esters, are particularly effective in reducing triglycerides. Omega-3 fatty acids and fenofibrates would best be reserved for those in whom lifestyle modification and treatment of secondary factors is inadequate.^19^^,^^55^

Although this patient's triglycerides are the primary concern, his LDL-C is well below 100 mg/dL, which may reduce the urgency for statin therapy. However, if triglycerides remain elevated and the patient's ASCVD risk increases (ie, a 10-year risk ≥5% or a diagnosis of diabetes), statin therapy should be initiated or intensified and would likely provide the greatest benefit for ASCVD risk reduction. In such cases, statins are recommended to target appropriate LDL-C or non-HDL-C thresholds, as they offer protection against both cardiovascular events and further elevations in triglycerides. In the setting of hypertriglyceridemia, LDL-C calculations may be less accurate (especially if the older Friedewald calculation is used), and a more accurate assessment of atherogenic risk may require the usage of non-HDL-C and/or ApoB to help decide on the intensity of lipid lowering.^5^^,^^6^ In these cases, it is important to emphasize the role of non-HDL-C and/or ApoB as elevations in either should prompt consideration of statin therapy prior to triglyceride-specific lowering agents such as omega-3 fatty acids or fibrates.

This patient's hypertriglyceridemia is likely driven by his prediabetes, diet, and sedentary lifestyle. Initial treatment should focus on lifestyle modification, including dietary changes and increased physical activity. Pharmacologic intervention, such as fenofibrate or prescription omega-3 fatty acids, may be necessary if triglycerides remain persistently elevated above 500 mg/dL despite addressing diet, weight, and glycemic control. Statin therapy could be considered if his ASCVD risk increases or if lipid targets are not met through other interventions. In addition, especially in South Asian individuals who may have premature coronary disease at lower risk factor thresholds, a CAC score may prove useful. This is overall consistent with the ACC ECDP on hypertriglyceridemia.^19^ The ESC guidance is more liberal in recommendations. For high-risk individuals with triglycerides >200 mg/dL, statins are recommended. In both primary and secondary prevention with LDL-C at treatment goal, fibrates are recommended at the lower level of 200 mg/dL.^20^ Similar to the ACC guidance, in high-risk individuals with persistent hypertriglyceridemia, icosapent ethyl is recommended, albeit starting at triglycerides >135 mg/dL.^20^ Similarly, the CCS guidelines recommend icosapent ethyl in patients with ASCVD with persistent hypertriglyceridemia >135 mg/dL with no specific recommendations for fibrates.^21^ Therefore, in this case, given that the patient was not high-risk, lifestyle changes would have been recommended by all guidelines. If his risk had increased, statin therapy would have been advised under all guidelines. However, there would have been a lower threshold under ESC guidelines to institute fibrates.

## Heterozygous FH in pregnancy

A 29-year-old woman with a history of severe hypercholesterolemia is referred to cardiology for further evaluation. She has a significant family history of premature coronary artery disease and hypercholesterolemia. Genetic testing confirms a single pathogenic mutation in the *LDLR* gene, diagnosing her with heterozygous familial hypercholesterolemia. She was started on high-intensity statin therapy, later combined with a PCSK9 monoclonal antibody due to persistently elevated LDL-C levels, which have remained above 100 mg/dL. Seven years later, she is contemplating pregnancy and seeks advice regarding the impact of her condition on pregnancy. At the time of consultation, her LDL-C is 107 mg/dL.

### Discussion

This case presents unique challenges related to managing FH during pregnancy, a period marked by significant physiological changes. Women with FH experience a rise in lipoprotein levels during pregnancy, including a 25% to 50% increase in cholesterol and a 2.5- to 3-fold increase in triglycerides, especially in the second and third trimesters. Importantly, in FH patients, the absolute increase in LDL-C can be substantial, exacerbating cardiovascular risk.^43^^,^^44^ Unfortunately, guidance is limited. The ACC ECDP does provide guidance that bile acid sequestrants may be used during pregnancy and that statins can be considered in high-risk individuals as below.^5^ The ESC guideline provides recommendations to avoid statins but notes bile acid sequestrants or lipoprotein apheresis can be considered in severe cases of hyperlipidemia^20^; the CCS guidelines have no recommendations on this subject.^21^

### Key considerations

**Figure 3:**
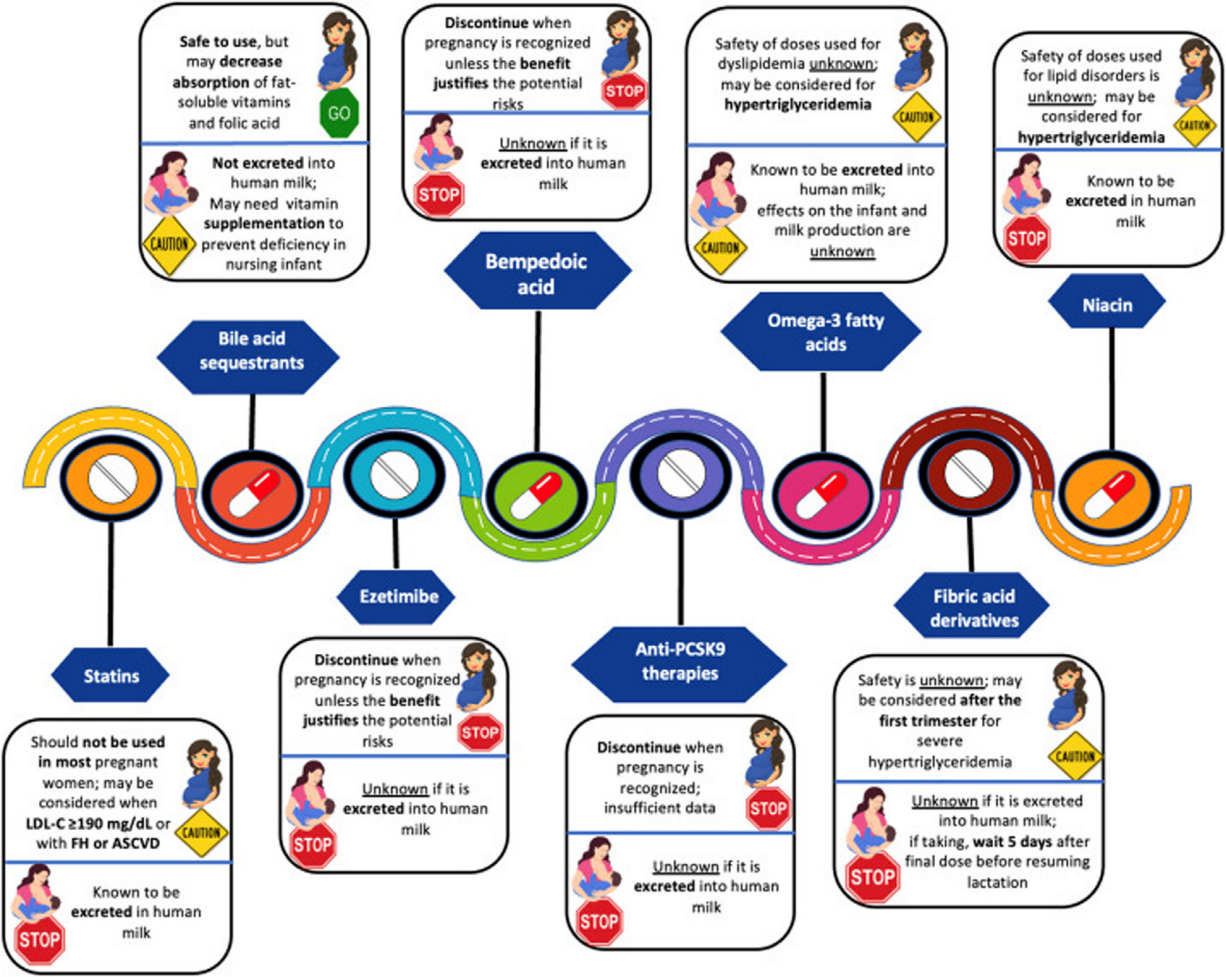
Overview of Lipid-Lowering Therapy in Women of Reproductive Age Adapted From Dyslipidemia Management in Women of Reproductive Potential: An Expert Clinical Consensus From the National Lipid Association Used with permission of the publisher.^43^
4) Lipid monitoring during pregnancy: While there are no universal guidelines for lipid screening during pregnancy, the ESC recommends monitoring lipids throughout pregnancy, particularly in high-risk patients.^44^ Lipid testing should be conducted at least once per trimester if no pharmacotherapy is used. In cases where severe dyslipidemia develops, bile acid sequestrants can be initiated. While statin therapy, particularly pravastatin, could be considered after the second trimester if LDL-C remains significantly elevated, and after an extensive risk/benefit discussion,^44^ this would best be done in conjunction with the patient's obstetrician and a lipid specialist. This should generally be reserved for those women at high risk, namely those with ASCVD and/or FH.

1) Pregnancy-related lipid changes: During pregnancy, lipid levels typically rise to support fetal development. In women with FH, these elevations are more pronounced due to an already elevated baseline LDL-C, leading to an increased risk of ASCVD progression. Therefore, careful preconception planning and management are essential.^45^
2) Preconception optimization: Counseling prior to conception is critical. This should include discussions about LLT discontinuation, partner lipid testing, and the potential for prenatal or preimplantation genetic testing. In women with FH, optimizing lipid levels and managing cardiovascular risk factors before pregnancy can mitigate ASCVD risk.^43^^,^^44^
3) Pharmacotherapy considerations during pregnancy (Figure 3): Statins, the mainstay of FH treatment, are generally avoided during conception, pregnancy, and breastfeeding due to concerns about teratogenicity. Although concerns have diminished with further research, statins are still not recommended unless absolutely necessary in high-risk cases (eg, homozygous FH or severe ASCVD), and even then, only after the first trimester. Statin therapy should ideally be discontinued 1 to 3 months before conception.^43^, ^44^, ^45^
  • Ezetimibe should also be avoided during pregnancy due to a lack of safety data.
  • PCSK9 inhibitors (monoclonal antibodies like evolocumab and alirocumab) cross the placenta and are not recommended during pregnancy or breastfeeding due to potential effects on neural tube development. These therapies should be discontinued prior to conception.
  • Bile acid sequestrants are considered safe during pregnancy as they are not systemically absorbed. However, these agents may impair the absorption of fat-soluble vitamins and folic acid, so supplementation may be required. In addition, their use may worsen hypertriglyceridemia, a potential concern in pregnancy.
  • Lipoprotein apheresis can be considered in high-risk patients, such as those with severe hypercholesterolemia, homozygous FH, or ASCVD. It is a safe and effective method for reducing LDL-C and Lp(a) levels during pregnancy, although its availability is limited.^59^
  • Inclisiran, an siRNA-based PCSK9 inhibitor, theoretically could offer benefits as it is rapidly cleared from the serum and provides durable LDL-C lowering over 6 months. However, this strategy has not been evaluated in pregnancy and would require further study.^43^, ^44^, ^45^

Managing FH in pregnancy requires a careful balance between minimizing maternal ASCVD risk and avoiding potential teratogenic effects of lipid-lowering medications. Preconception counseling, regular lipid monitoring during pregnancy, and the use of bile acid sequestrants or lipoprotein apheresis in high-risk cases are key strategies. Statin therapy may be cautiously reintroduced after the second trimester in select high-risk individuals, but generally, most LLTs are avoided until after pregnancy and breastfeeding. Given this patient's diagnosis of heterozygous FH and her elevated baseline LDL-C, individualized management is crucial. She should discontinue statin and PCSK9 inhibitor therapy 1 to 3 months before attempting conception. A review of cardiovascular risk and atherosclerotic plaque burden using coronary calcium scoring or a coronary computed tomography angiogram may be beneficial in further stratifying her risk prior to conception.^43^^,^^45^ Lipid levels should be monitored regularly. If LDL-C becomes significantly elevated, bile acid sequestrants are the safest pharmacologic option. Statins can be cautiously considered after the second trimester in women at high cardiovascular risk, such as those with severe hypercholesterolemia or established ASCVD. After delivery, statin therapy can be resumed. However, breastfeeding women should avoid statins, ezetimibe, and PCSK9 inhibitors, as they are contraindicated during lactation. Bile acid sequestrants can be continued if necessary.

## Elevated Lipoprotein(a) with CAC score of 0 AU

A 43-year-old African American male with a history of hyperlipidemia and hypertension presents for his annual exam. His fasting lipid panel reveals:

• LDL-C: 139 mg/dL
• HDL-C: 34 mg/dL
• Triglycerides: 123 mg/dL
• Total cholesterol: 198 mg/dL

He has a blood pressure of 126/79 mm Hg on amlodipine 5 mg and a hemoglobin A1c of 4.9% and is a never-smoker. There is a significant family history of premature coronary artery disease. The primary care physician calculates a 10-year ASCVD risk using the PCE, resulting in a 6.7% risk estimate (PREVENT 10-year risk of CVD 2.9%, ASCVD 2.0%). Given the family history of premature coronary disease, an Lp(a) level was measured, returning a value of 215 nmol/L—well above the threshold for elevated risk. The patient expressed uncertainty about starting statin therapy. A CAC score was obtained, returning 0 AU. The patient was referred to cardiology for further discussion on management.

### Discussion

This patient presents with a significantly elevated Lp(a) level, a recognized risk-enhancing factor for ASCVD.^22^ While the patient's CAC score of 0 AU is reassuring, indicating no calcified plaque and a low short-term cardiovascular event risk, the elevated Lp(a) remains a concern. Lp(a) is associated with noncalcified, potentially unstable atherosclerotic plaques that may not be detected by CAC scoring, leaving the patient at elevated risk of long-term cardiovascular events, especially as the minority of men younger than age 45 years have coronary calcification.^60^^,^^61^

### Key considerations

• Role of CAC score and Lp(a) in risk assessment: The absence of CAC in this patient reduces his short-term risk of ASCVD events, as individuals with a CAC score of 0 generally have a low risk of cardiovascular events over the next 5 to 10 years.^62^ However, elevated Lp(a) is associated with the development of noncalcified, vulnerable plaques, which may pose a risk that CAC scoring alone cannot capture. Lp(a) is an independent predictor of ASCVD, even in the absence of calcified plaque. Therefore, it warrants careful consideration in long-term risk stratification.^60^^,^^61^
• Additional risk factors to consider: Given the borderline 10-year ASCVD risk (6.6%) and the presence of 2 risk-enhancing factors—elevated Lp(a) and a family history of premature coronary artery disease (CAD)—additional markers could be considered to further refine risk^18^:
  1) High-sensitivity C-reactive protein (hs-CRP): Elevated levels of hs-CRP can indicate systemic inflammation, which may provide insights into the patient's atherosclerotic risk.
  2) ApoB: Measuring ApoB could give a more accurate assessment of atherogenic lipoprotein particles, complementing traditional LDL-C measurements.
  3) Ankle-Brachial Index: A simple, noninvasive test that may identify subclinical peripheral arterial disease.
  4) Metabolic syndrome: Assessing for components of metabolic syndrome, such as increased waist circumference or elevated fasting glucose, could help guide management.
• Management strategy: Despite the low CAC score, the patient's elevated Lp(a) and family history suggest an elevated lifetime risk of ASCVD, justifying consideration of pharmacotherapy. The primary goals are to reduce LDL-C levels and overall cardiovascular risk.

### Pharmacologic therapy

• Statin therapy: Initiation of moderate-intensity statin therapy is reasonable, with the aim of lowering LDL-C by at least 30% or achieving LDL-C levels below 100 mg/dL. Statins are the first-line treatment in this case, given their proven efficacy in reducing ASCVD risk, even in patients with normal or low CAC scores.
• Lp(a) monitoring: While elevated Lp(a) is a risk-enhancing factor, it does not necessitate serial monitoring after the initial measurement. The primary focus of treatment is the management of modifiable risk factors, particularly LDL-C, as current therapies do not specifically target Lp(a).^18^^,^^27^

### Lifestyle modifications

In addition to pharmacotherapy, lifestyle interventions should be emphasized. These include:

• Heart-healthy diet: Adopting a diet low in saturated fats and refined carbohydrates, while rich in fruits, vegetables, whole grains, and healthy fats, is essential.
• Physical activity: Encouraging regular physical activity, aiming for at least 150 minutes of moderate-intensity aerobic exercise per week, can help improve lipid profiles and overall cardiovascular health.^18^^,^^27^^,^^39^

Although the patient's CAC score of 0 AU suggests a low short-term risk of cardiovascular events, the elevated Lp(a) level and family history of premature CAD indicate an elevated long-term ASCVD risk. Initiating moderate-intensity statin therapy, along with lifestyle modifications, is recommended to reduce LDL-C and mitigate the patient's lifetime ASCVD risk. Regular monitoring of lipid levels and continued cardiovascular risk assessment are critical to ensure appropriate management and adherence to therapy. A fasting lipid panel should be repeated 4 to 12 weeks after initiating statin therapy to assess the patient's response. The goal is to achieve at least a 30% reduction in LDL-C from baseline or an LDL-C level below 100 mg/dL. Further follow-up visits should assess adherence to therapy and lifestyle changes, as well as monitor for any adverse effects of the medication. This approach of using Lp(a) as a risk-enhancing factor is consistent with AHA/ACC guidance.^18^ Furthermore, de-risking patients with a CAC of 0 is consistent with the ACC ECDP although no specific note is made of Lp(a) and CAC of 0.^5^ Similarly, the ESC and CCS guidelines discuss Lp(a) as a risk-enhancing feature but do not discuss a role for CAC testing in this setting.^20^^,^^21^ No guideline provides specific treatment recommendations based on elevated Lp(a).

## Statin intolerance

A 59-year-old Caucasian male with a history of hyperlipidemia and well-controlled hypertension presents for his yearly exam. His fasting lipid panel shows:

• LDL-C: 145 mg/dL
• HDL-C: 36 mg/dL
• Triglycerides: 137 mg/dL
• Total cholesterol: 208 mg/dL

His blood pressure is 122/73 mm Hg on lisinopril 10 mg, and his hemoglobin A1c is 5.2%. He is a never-smoker, with no significant family history of premature CAD. His primary care physician calculates a 10-year ASCVD risk of 11.3% using the PCE (PREVENT 10-year risk of CVD 8.6%, ASCVD 5.5%). After discussing the risks and benefits of therapy, the patient was prescribed atorvastatin 20 mg, a moderate-intensity statin. Approximately 8 weeks later, he reports muscle aches in his thighs. Despite reducing the atorvastatin dose to 10 mg, his symptoms persist, and the statin is eventually discontinued, leading to symptom resolution. A trial of rosuvastatin 5 mg also results in muscle aches.

### Discussion

**Figure 4:**
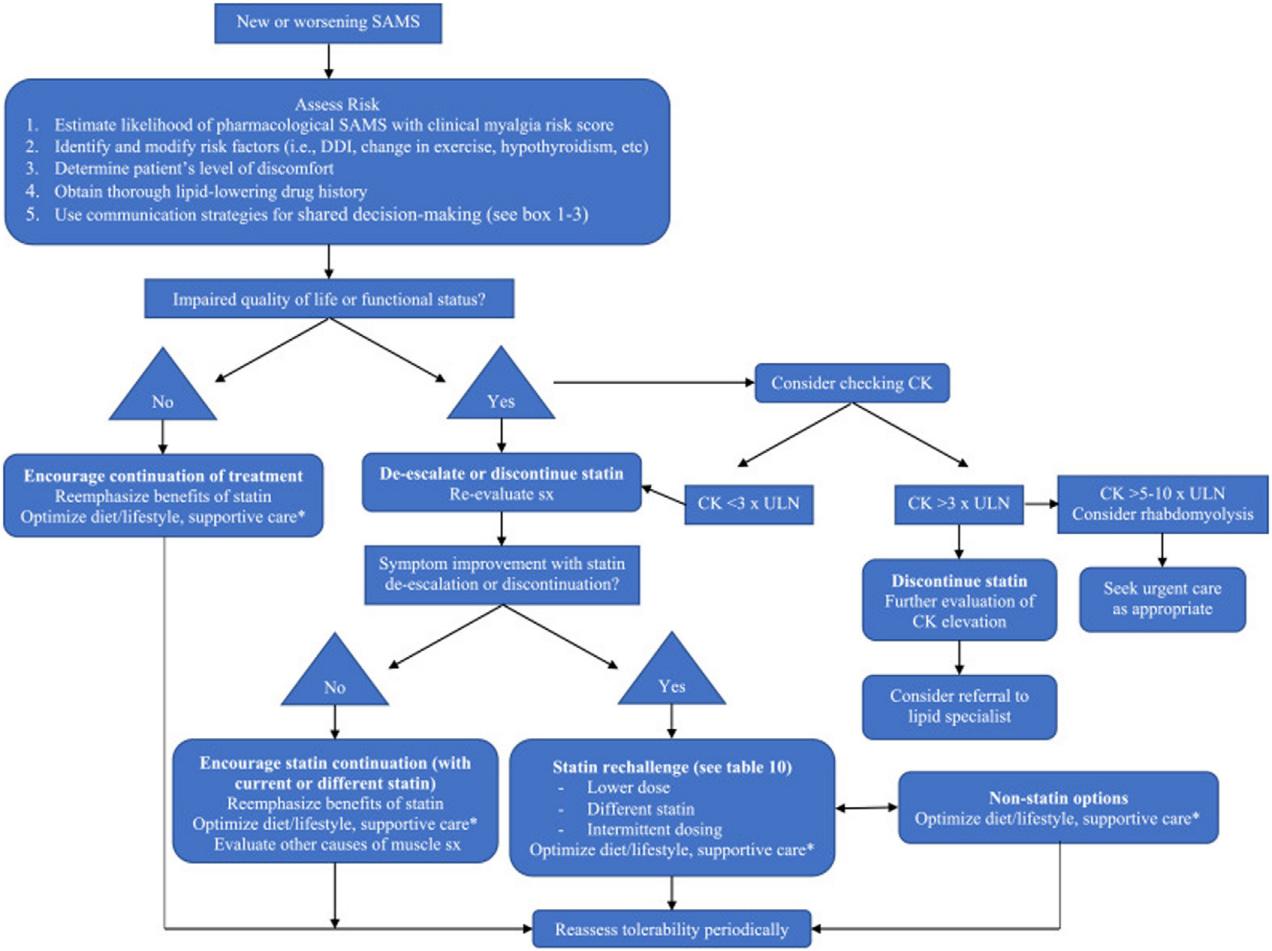
Overview of the Assessment and Management of Statin-Associated Muscle Symptoms Adapted From Assessment and Management of Statin-Associated Muscle Symptoms (SAMS): A Clinical Perspective From the National Lipid Association Used with permission of the publisher.^13^

This case highlights the challenge of managing statin intolerance, specifically SAMS, which is the most common side effect of statin therapy and the leading cause of discontinuation. SAMS refers to a spectrum of muscle symptoms, most commonly bilateral myalgias in large muscle groups, that are temporally related to statin therapy. Symptoms typically occur 4 to 12 weeks after initiation and resolve within 2 to 4 weeks of stopping the statin. The majority of SAMS cases do not involve creatine kinase (CK) elevation, and routine CK testing is not generally recommended^13^^,^^14^ (Figure 4).

### Key considerations

• Assessment of SAMS: The first step in managing suspected SAMS is assessing the likelihood of true statin intolerance using a validated tool, such as the National Lipid Association Statin Myalgia Clinic Index. Secondary causes of muscle symptoms should also be evaluated, including^14^^,^^61^:
  1) Drug interactions: Medications that interfere with statin metabolism can increase the risk of SAMS.
  2) Hypothyroidism: Although hypothyroidism can contribute to myalgias, thyroid function should be normal before assuming SAMS is the cause.
  3) Vitamin D deficiency: Low vitamin D levels can cause muscle pain, but supplementation has not been definitively shown to affect SAMS.
  4) Coenzyme Q10 (CoQ10) supplementation: While anecdotal evidence suggests CoQ10 may improve SAMS, data are discordant, and current guidelines do not recommend its routine use. However, patients may choose to try CoQ10 if they wish to continue statin therapy.
• Management of statin intolerance^13^^,^^63^:
  1) Mild symptoms: If the patient's muscle symptoms are mild and do not impair function or quality of life, the statin can be continued, with supportive measures such as stretching, hydration, and sleep hygiene. If symptoms persist, de-escalation of the statin dose or temporary discontinuation with a rechallenge is a reasonable approach.
  2) Severe symptoms: If symptoms significantly impact the patient's quality of life, discontinuation of the statin is appropriate. Rechallenge with a lower dose of the same or an alternative statin can be attempted after a 2- to 4-week washout period. Statins like pravastatin or fluvastatin may be better tolerated as they are less likely to cause muscle symptoms.
• CK testing: Routine CK testing is not indicated for mild SAMS, as most patients do not have elevated CK levels. However, CK testing is warranted if the patient experiences severe pain, objective muscle weakness, or if CK elevation is suspected. If CK levels exceed 3 × the upper limit of normal, the statin should be discontinued. CK elevations >5-10× upper limit of normal require urgent evaluation for rhabdomyolysis.^13^^,^^14^
• Pharmacologic alternatives to statins: If statin intolerance is confirmed, alternative LLTs should be considered. The 2022 ACC/AHA ECDP for nonstatin therapies outlines several options^5^:
  1) Ezetimibe: As a first-line nonstatin agent, ezetimibe reduces LDL-C by inhibiting cholesterol absorption in the intestine. It is well-tolerated and can be used alone or in combination with the lowest tolerable dose of a statin.
  2) Bempedoic acid: The CLEAR Outcomes trial demonstrated that bempedoic acid is an effective option for statin-intolerant patients.^64^ Bempedoic acid, in combination with ezetimibe, has LDL-C-lowering efficacy comparable to moderate-intensity statins. This therapy is particularly useful for high-risk primary prevention patients, such as those with diabetes or elevated CAC scores.
  3) Bile acid sequestrants: Although second-line agents, bile acid sequestrants can be considered if the patient is unable to tolerate both statins and ezetimibe. However, they may cause gastrointestinal side effects and have limited tolerability.
  4) PCSK9 inhibitors: While PCSK9 inhibitors (eg, evolocumab, alirocumab) are not recommended for most primary prevention patients, they can be considered in very-high-risk individuals (eg, those with a CAC score >1,000 AU or severe dyslipidemia). These agents are more frequently used in secondary prevention or patients with LDL-C >190 mg/dL.
  5) Inclisiran: Although inclisiran (a siRNA-based PCSK9 inhibitor) may offer long-term LDL-C reduction with less-frequent dosing, it lacks cardiovascular outcomes data in statin-intolerant patients and should be used cautiously.
• Secondary prevention considerations: If this patient were being treated for secondary prevention (ie, if he had known ASCVD or severe hyperlipidemia with LDL-C >190 mg/dL), the need for LDL-C lowering would be more urgent. In such cases, more aggressive therapy with statins (if tolerable) or nonstatin agents like PCSK9 inhibitors would be warranted. The goal in secondary prevention is to achieve an LDL-C of <70 mg/dL or a ≥50% reduction from baseline.

In this case, the patient's muscle symptoms are consistent with SAMS. A stepwise approach to statin rechallenge and dose de-escalation should be attempted. If statin intolerance persists, nonstatin therapies such as ezetimibe or bempedoic acid should be initiated to achieve appropriate LDL-C reduction. For primary prevention, a target LDL-C reduction of 30% or a level <100 mg/dL is appropriate, while in secondary prevention, more aggressive targets would be required. Regular follow-up and monitoring are essential to ensure efficacy and adherence to therapy. As noted earlier, this is consistent with the ACC ECDP.^5^ The ESC and CCS guidelines are more limited in discussion of statin intolerance and merely note that ezetimibe and PCSK9 monoclonal antibodies are options for treatment depending on clinical situation and cost.^20^^,^^21^

## Conclusions

Despite significant advances in the prevention and treatment of ASCVD, it remains a major global cause of morbidity and mortality. Central to ASCVD prevention is effective lipid-lowering, which reduces both the onset of disease and the risk of recurrent cardiovascular events in those with established disease. However, translating these evidence-based strategies into routine clinical practice presents several challenges. Achieving guideline-directed lipid management can be complicated by factors such as patient intolerance, individual variability in response to therapy, and the presence of risk-enhancing factors that are not always neatly addressed by current guidelines.

Moreover, many commonly encountered clinical scenarios—as depicted in the aforementioned case examples—require nuanced, patient-centered approaches that extend beyond standard recommendations. The rapidly evolving landscape of LLTs, including the development of novel agents such as PCSK9 inhibitors, bempedoic acid, and inclisiran, provides clinicians with new tools to tailor treatments, but also necessitates a deeper understanding of when and how to use these therapies effectively. This review has aimed to bridge the gap between guideline-directed care and real-world clinical complexities, offering practical insights into managing patients with challenging lipid disorders. By contextualizing current recommendations and highlighting emerging treatment options, we hope to empower clinicians to make informed, individualized treatment decisions that improve outcomes for their patients. As the field of lipidology continues to evolve, it is crucial that healthcare providers employ both established and emerging therapies to reduce ASCVD risk and enhance patient care.

## Funding support and author disclosures

Dr Sharma has received a grant from the 10.13039/100000968American Heart Association (979462), unrelated to the present work. Dr Mehta has received research grants from 10.13039/100008272Novartis and 10.13039/100002429Amgen, unrelated to the present work. P.N. reports research grants from Allelica, 10.13039/100002429Amgen, Apple, 10.13039/100008497Boston Scientific, 10.13039/100001127Genentech/Roche, and 10.13039/100008272Novartis; personal fees from Allelica, Apple, AstraZeneca, Blackstone Life Sciences, Creative Education Concepts, CRISPR Therapeutics, Eli Lilly & Co, Foresite Labs, Genentech/Roche, GV, HeartFlow, Magnet Biomedicine, Merck, and Novartis; scientific advisory board membership of Esperion Therapeutics, Preciseli, and TenSixteen Bio; scientific co-founder of TenSixteen Bio, equity in MyOme, Preciseli, and TenSixteen Bio; and spousal employment at Vertex Pharmaceuticals, all unrelated to the present work. Dr Nasir is on the advisory board of Novo Nordisk, Novartis, Esperion, Merck Sharp and Dohme, and ER Squib and Sons; and reports grants from 10.13039/100000002National Institutes of Health, 10.13039/100006093Patient-Centered Outcomes Research Institute, 10.13039/100008272Novartis, and 10.13039/501100022336Esperion, all unrelated to the present work. Dr Shapiro has received institutional grants from Amgen, Arrowhead, Boehringer Ingelheim, 89Bio, Esperion, 10.13039/100008272Novartis, Ionis, Merck, New Amsterdam, Lilly, and Cleerly; has participated in scientific advisory boards with Amgen, Agepha, Ionis, Novartis, New Amsterdam, and Merck; and has served as a consultant for Ionis, Novartis, Regeneron, Aidoc, Shanghai Pharma Biotherapeutics, Kaneka, Novo Nordisk, Arrowhead, and Tourmaline, all unrelated to the present work. All other authors have reported that they have no relationships relevant to the contents of this paper to disclose.
